# The conserved protein kinase-A target motif in synapsin of *Drosophila *is effectively modified by pre-mRNA editing

**DOI:** 10.1186/1471-2202-7-76

**Published:** 2006-11-14

**Authors:** Sören Diegelmann, Vanessa Nieratschker, Ursula Werner, Jürgen Hoppe, Troy Zars, Erich Buchner

**Affiliations:** 1 Lehrstuhl für Genetik und Neurobiologie, Theodor Boveri Institut für Biowissenschaften der, Universität Würzburg, Würzburg, Germany; 2 Lehrstuhl für Physiologische Chemie, Theodor Boveri Institut für Biowissenschaften der, Universität Würzburg, Würzburg, Germany; 3 Division of Biological Sciences, University of Missouri-Columbia, Columbia, USA

## Abstract

**Background:**

Synapsins are abundant synaptic vesicle associated phosphoproteins that are involved in the fine regulation of neurotransmitter release. The *Drosophila *member of this protein family contains three conserved domains (A, C, and E) and is expressed in most or all synaptic terminals. Similar to mouse mutants, synapsin knock-out flies show no obvious structural defects but are disturbed in complex behaviour, notably learning and memory.

**Results:**

We demonstrate that the N-terminal phosphorylation consensus motif RRxS that is conserved in all synapsins investigated so far, is modified in *Drosophila *by pre-mRNA editing. In mammals this motif represents the target site P1 of protein kinase A (PKA) and calcium/calmodulin dependent protein kinase I/IV. The result of this editing, by which RRFS is modified to RGFS, can be observed in cDNAs of larvae and adults and in both isolated heads and bodies. It is also seen in several newly collected wild-type strains and thus does not represent an adaptation to laboratory culture conditions. A likely editing site complementary sequence is found in a downstream intron indicating that the synapsin pre-mRNA can form a double-stranded RNA structure that is required for editing by the adenosine deaminase acting on RNA (ADAR) enzyme. A deletion in the *Drosophila Adar *gene generated by transposon remobilization prevents this modification, proving that the ADAR enzyme is responsible for the pre-mRNA editing described here. We also provide evidence for a likely function of synapsin editing in *Drosophila*. The N-terminal synapsin undeca-peptide containing the genomic motif (RRFS) represents an excellent substrate for in-vitro phosphorylation by bovine PKA while the edited peptide (RGFS) is not significantly phosphorylated. Thus pre-mRNA editing by ADAR could modulate the function of ubiquitously expressed synapsin in a cell-specific manner during development and adulthood.

**Conclusion:**

Similar to several other neuronal proteins of *Drosophila*, synapsin is modified by ADAR-mediated recoding at the pre-mRNA level. This editing likely reduces or abolishes synapsin phosphorylation by PKA. Since synapsin in *Drosophila *is required for various forms of behavioural plasticity, it will be fascinating to investigate the effect of this recoding on learning and memory.

## Background

After the discovery of RNA editing in the kinetoplastid of typanosomes nearly two decades ago [1], similar processes have been observed for a large number of genes from different species. RNA editing modifies the information encoded by the genomic DNA post-transcriptionally at the RNA level [2,3]. Most examples of nuclear pre-mRNA editing in higher eukaryotes employ adenosine to inosine (A-to-I) conversion which is generally discovered by an adenosine vs. guanosine discrepancy between genomic and cDNA sequences because inosine and guanosine both pair with cytidine during cDNA synthesis by reverse transcriptase. The conversion is catalyzed by adenosine deaminases acting on RNA (ADARs). The hydrolytic deamination by the enzyme requires a double-stranded RNA structure formed by the editing site and an intronic region that contains an "editing site complementary sequence" (ECS) motif [4-6].

In *Drosophila melanogaster *deletions in the only gene (*Adar*) with homology to ADARs cause severe behavioural abnormalities and neurological symptoms including temperature-sensitive paralysis, uncoordinated movements, and tremors [7]. Many known target genes of ADAR in *Drosophila *are involved in fast electrical and chemical neurotransmission, indicating that RNA editing by ADAR in this species may be of particular relevance for nervous system function [8,9].

Synapsins constitute a family of highly conserved proteins of the nerve terminal. In vertebrates they have been shown to bind to synaptic vesicles (SV) and cytoskeletal elements in a phosphorylation-dependent manner [10-12]. The present hypothesis on synapsin function proposes that at rest synapsins attenuate neurotransmitter release by binding SVs of the reserve pool to actin filaments of the cytoskeleton. Synaptic activity leads to calcium influx and phosphorylation of synapsin by calcium dependent kinases, which reduces its affinity to SVs and actin. This causes the release of the SVs from the cytoskeleton such that they can move to the active zone for exocytosis. Support for this hypothesis has been obtained by in vivo imaging of the redistribution of GFP-labelled synapsin during stimulation [13,14]. However, synapsins have also been proposed to be involved in various other neuronal activities, including neurite elongation, synaptogenesis, synaptic maturation, and synaptic plasticity [15-21]. Similar to *synapsin *null mutant flies, triple knock-out mice, in which all three *synapsin *genes found in mammals have been inactivated, are viable and fertile but exhibit various behavioural defects. Cultured neurons from these mice show differential synaptic alterations at excitatory and inhibitory synapses [12,22]. At the calyx of Held synapse in the brainstem of mice it was recently demonstrated that synapsins 1 and 2 are only required for enhancing vesicle release probability during high frequency stimulation [23]. Interestingly, a nonsense mutation in the human *synapsin-I *gene has been identified as the likely cause for a complex behavioural phenotype, displaying epilepsy, learning difficulties, and aggressive behaviour [24].

Invertebrate synapsins share with vertebrate synapsins three conserved regions, termed domains A, C, and E [25]. In *Drosophila *a single *synapsin *gene (*Syn*) is found, which has been cloned and molecularly characterized [26]. Targeted deletion of this gene does not impair basic synaptic structure or function but leads to a variety of defects in complex behaviour, in particular in tasks involving learning and memory [27,28]. When the genomic sequence of the *synapsin *gene became available [29], we noted a single base substitution (A to G) in several independently cloned cDNAs within the codons for the A-domain sequence RRFS that conforms to the RRxS phosphorylation motif conserved in all known synapsins [25]. We show that this A vs. G discrepancy between genomic and cDNA sequences is found in all wild-type strains investigated and during all tested stages of development. The structural requirements for A to I editing by the ADAR enzyme are fulfilled. Since the *Adar *mutants described earlier [7,8] were not available we created a new deficiency allele of the *Adar *gene and demonstrate that the ADAR enzyme is required for the observed editing of the conserved kinase recognition motif in domain A of *Drosophila *synapsin. In mammals and in *Aplysia *this motif has been identified as a target site for cAMP-dependent protein kinase (PKA) and calcium/calmodulin dependent protein kinase I/IV. Phosphorylation of *Aplysia *synapsin at this site by PKA has been suggested to play a role in regulation of neurotransmitter release [30] and short-term plasticity [31]. In developing as well as in adult mammalian neurons phosphorylation by PKA regulates the rate of synaptic vesicle recycling [32-34]. Here we observe that an undeca-peptide containing the genome-encoded N-terminal sequence of *Drosophila *synapsin is readily phosphorylated by bovine PKA, whereas the cDNA-encoded undeca-peptide is not efficiently phosphorylated by this enzyme.

## Results

### Examination of the boundaries of intron 4 in the *Drosophila synapsin *gene

**Figure 1:**
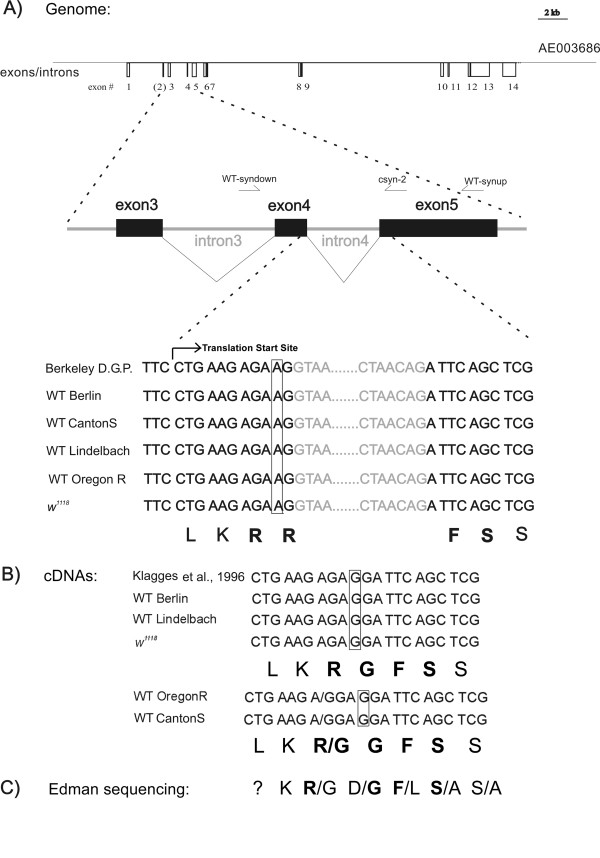
**Structure of the *Drosophila synapsin *gene and genomic and cDNA sequences near the protein kinase target motif, including the encoded amino acids**. The base modified by RNA editing is boxed. (A). Schematic representation of the *synapsin *locus of *Drosophila melanogaster *showing the exon-intron structure of the gene. Underneath, a close-up view of exons 4 and 5 with the sequence from the Berkeley *Drosophila *Genome Project and five different wild-type strains at this site is depicted. The encoded amino acids are indicated under each codon by the one letter code, bold letters mark the kinase target motif RRFS. (B). cDNA sequences from an earlier publication [26] and five different wild-type lines. The region surrounding the phosphorylation target motif in the synapsin cDNA was amplified by nested RT-PCR and sequenced. The relevant codons and the corresponding amino acids are shown. The genomic kinase target motif RRFS is modified to RGFS at the cDNA level (bold letters). In two wild-type strains partial editing also modifies the first arginine of the motif. (C). Amino acid sequence obtained by Edman degradation of purified *Drosophila *synapsin. At most positions two amino acids were detected, resulting from an unknown contamination (from [27]). The sequence is compatible only with the cDNA encoded version of the kinase target motif (bold letters).

When the *Drosophila *genome became available from the Berkeley *Drosophila *Genome Project (BDGP) [29] a clear single base discrepancy between the genomic sequence and the previously published sequence of a cDNA from a head cDNA library [26] was noted. We therefore wanted to test whether the affected region was polymorphic by amplifying and sequencing the corresponding genomic region in five different laboratory wild-type strains. In all cases the genome project sequence was verified (Fig. 1A) which predicts an open reading frame containing an RRFS motif compatible with the consensus pattern for phosphorylation by PKA, [RK](2)-x-[ST] (Prosite). Thus it seemed that the pattern RRxS, which is found in the N-terminal A-domain of all known synapsin isoforms of both vertebrates and invertebrates, is also conserved in the *Drosophila *synapsin. The published cDNA sequence [26], however, codes for RGFS at this site due to a single A to G transition between genome and cDNA (Fig. 1B). The sequence RGFS is supported by N-terminal Edman degradation (Fig. 1C) of immuno-affinity purified synapsin protein (apparently contaminated by another polypeptide) from *Drosophila *heads [27]. Thus both, amino acid sequencing and cDNA translation suggest that the PKA consensus site is modified in all or the majority of synapsin protein from adult *Drosophila *heads. To further investigate this discrepancy between the cloned cDNA and genomic sequence, we examined additional cDNAs from the five laboratory wild-type strains. The sequences were produced by reverse transcription PCR using poly-A^+ ^mRNA as a template (Fig. 1B). In all five wild-type strains the A to G base substitution that leads to the observed amino acid exchange at the second position in the kinase consensus sequence was detected. Since in these experiments RNA was isolated from flies of all ages and no unedited cDNAs were observed we conclude that synapsin editing does not significantly vary with age of the flies. In two strains, wild-type OregonR and CantonS, the A to G substitution was accompanied by two overlapping peaks (A/G) of similar height in the sequencing record (Fig. 1B, see also Fig. 4) within the first triplet of the consensus, leading to an additional replacement of the first arginine of the consensus by glycine presumably in about half the proteins. We thus conclude that extensive editing occurs in this region of the synapsin mRNA during RNA maturation.

### RNA editing in newly established wild-type lines

**Figure 2:**
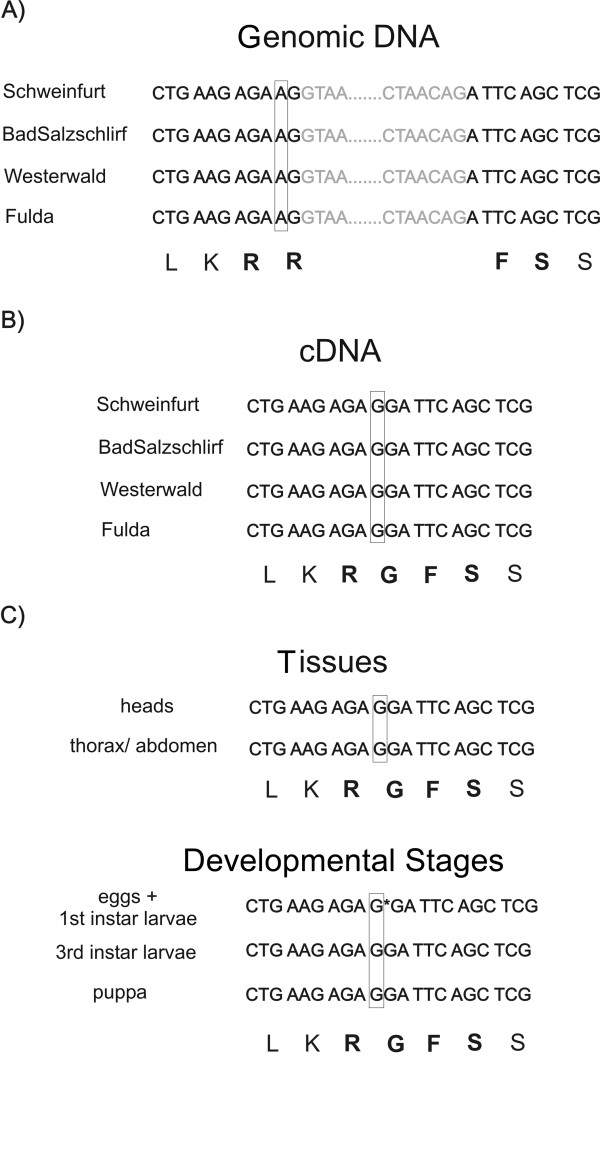
**Editing in newly collected flies, in different tissues, and at different development stages**. The base modified by RNA editing is boxed. (A). The genomic sequence at the boundaries of intron 4 from four different wild-type strains newly collected in central Germany. The black letters give sequences from exons 4 and 5, grey letters belong to intron 4. The corresponding amino acids are shown underneath the codons. The protein kinase target motif at the genomic sequence is in all cases RRFS (bold letters). (B). cDNA sequences from the lines in A). In all strains editing of a genomic adenosine to a guanosine in the cDNA leads to the modified motif RGFS (bold letters). (C). cDNAs from different tissues and different development stages. In both head and body homogenates the RNA modification was detected. RNA editing is also found during all development stages. In all cases RNA editing appears to modify all pre-mRNA except in the fraction of eggs plus first instar larvae where an adenosine peak overlaps in the sequence record with the G (asterisk).

To test whether the high efficiency of RNA editing of synapsin pre-mRNA might be an adaptation to the laboratory environment during the decade-long maintenance of the stocks under unnatural conditions, we collected wild flies in different regions of central Germany. The newly established *Drosophila melanogaster *lines were subjected to the same genomic DNA and cDNA sequence analysis as above. In all four strains we discovered the same genomic sequence as in our laboratory lines, in agreement with the sequence from the BDGP (Fig. 2). Sequencing of cDNAs from these new lines again showed in all cases that the second arginine in the kinase consensus motif has been changed to a glycine by the same A to G substitution during mRNA maturation. The first arginine codon of the kinase recognition site was not affected in these lines.

### RNA editing is observed in different tissues and at several developmental stages

Synapsins are expressed in the entire nervous system of the fly. To find out if RNA editing differs in thoracic and abdominal ganglia from that observed in the brain we separated head and thorax/abdomen and repeated the above experiments for each homogenate separately. In both tissues we found the edited cDNA (Fig. 2). In third instar larvae and in pupae we also found the A to G exchange. Only in a fraction which combined cDNAs from eggs and first instar larvae, evidence for a non edited form of the mRNA could be detected. In this case two overlapping peaks for A and G in the second arginine codon of the kinase site were obtained.

### No editing is observed at a second protein kinase A consensus motif of synapsin

**Figure 3:**
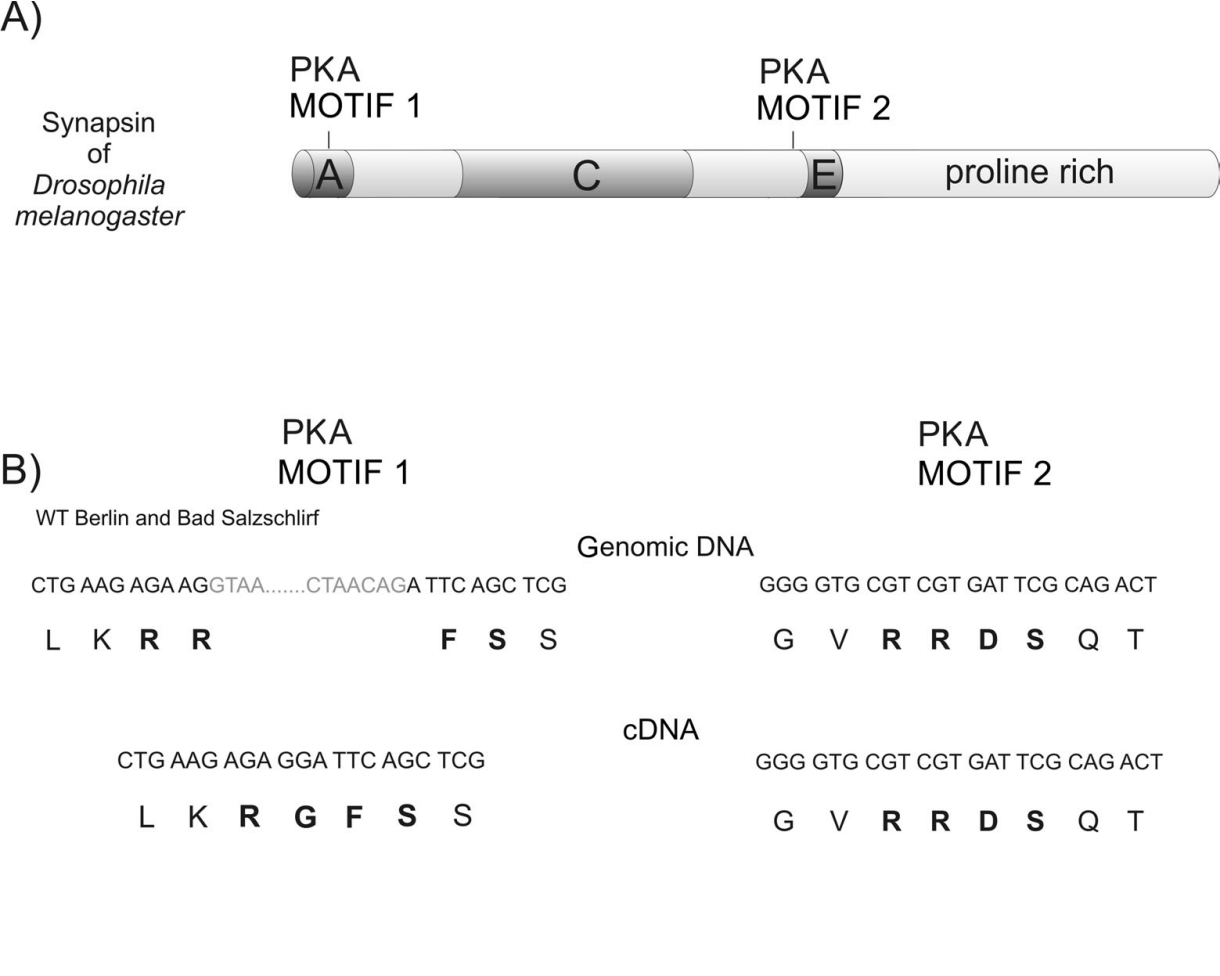
**No RNA editing at the second kinase consensus motif in *Drosophila *synapsin**. (A). Schematic representation of the domain structure of the 143 kDa *Drosophila *synapsin isoform. The A- C- and E-domains are conserved between vertebrates and invertebrates. The *Drosophila *protein contains two consensus motifs for PKA, one in the A-domain and the other near the E-domain. (B). Comparison of genomic and cDNA sequences of the regions coding for the two kinase target consensus motifs of two wild-type strains. Only the first consensus sequence is modified by RNA editing.

Within the *Drosophila *synapsin protein there is another potential recognition site for PKA/CamK-I/IV. This site (RRDS) lies adjacent to the E-domain of the protein (Fig. 3A) and is encoded by exon 13. To investigate whether this site is also edited we examined genomic DNA and the cDNA sequences for this site in two different wild-type strains, the laboratory line wild-type Berlin and the newly collected strain Bad Salzschlirf. Here the AGA codons for the first and the second arginine were not modified by RNA editing (Fig. 3B).

### Jump-out mutagenesis of the Adar gene using P-element line P{GT1}Adar

**Figure 4:**
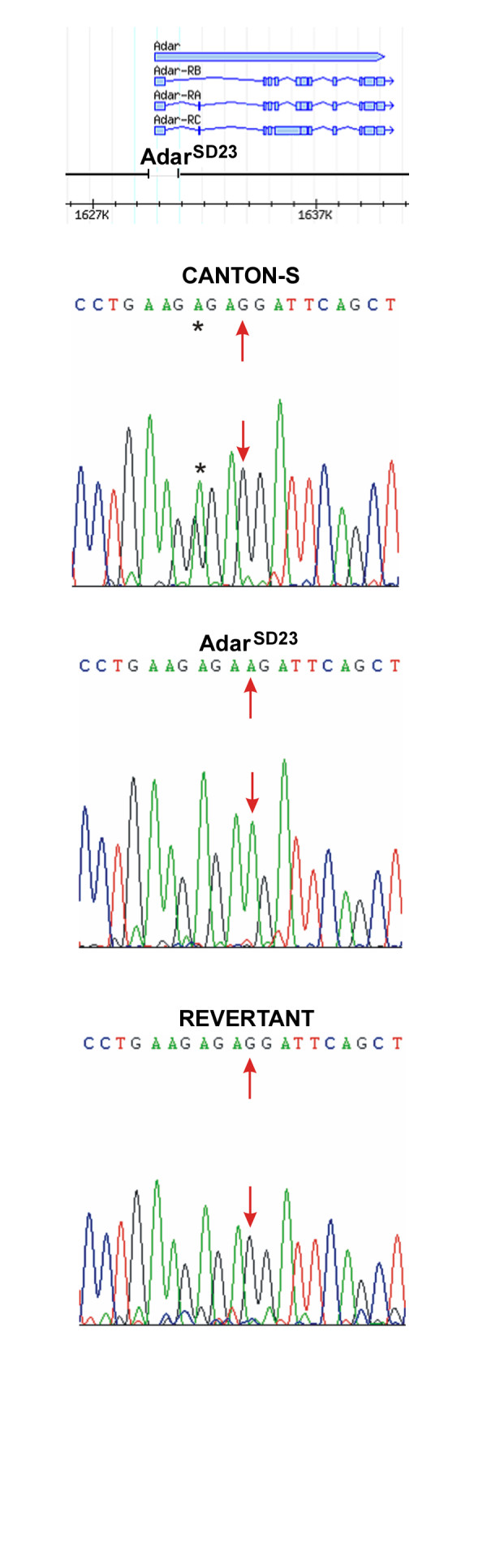
**Map of the new deficiency allele (*Adar*^*SD23*^) for the *Adar *gene and sequencing records of wild-type Canton-S, the mutant, and a revertant line from the same genetic background**. Exon-intron structure of the *Adar *gene with base ruler (in kb, modified from FlyBase) and position of the 1,197 bp deficiency in the *Adar*^*SD23 *^allele (top panel). The arrows in the lower three panels point to the base of the second codon of the kinase target motif that is edited in all wild-type strains investigated and in the revertant, but not in the mutant. In the wild type Canton-S an additional partial editing is observed in the first arginine codon indicated by overlapping peaks in the record (asterisks).

White-eyed jump-out lines (cf. Methods) which had lost the P-element were characterized by PCR and sequencing. Line #42 suffered a deletion of 736 bp confined to intron 1, is homozygous viable and displays no obvious phenotype. Line #13 contains an insertion of 31 bp (remnant of the P-element) in intron 1 and is considered a revertant. Line #23 suffered a deletion of 1,197 bp including the entire first exon and 210 bp of 5' regulatory sequences (promotor) and 513 bp of the first intron (Fig. 4, top panel). This latter line is semi-lethal, and homozygous escapers display the temperature-sensitive paralytic phenotype described for *Adar *null mutants [7]. We conclude that line #23 (*Adar*^*SD23*^) represents a new hypomorphic or null allele for the *Adar *gene.

### The kinase target motif in the synapsin "A" domain is not edited in the *Adar*^*SD23 *^mutant

In an additional set of experiments the synapsin cDNA sequence of wild type (Canton-S), the homozygous mutant (*Adar*^*SD23*^), and the jump-out revertant (line #13) was obtained by RT-PCR for the region coding for the kinase target motif (Fig. 4). Clearly, the discrepancy between genomic and cDNA sequence (arrow) is abolished only in the mutant, demonstrating that the pre-mRNA editing analyzed here depends on the presence of the intact *Adar *gene. In addition, the partial editing of the first arginine codon in the strain Canton-S is verified (asterisk).

### The edited form of synapsin is not phosphorylated efficiently by PKA

**Figure 5:**
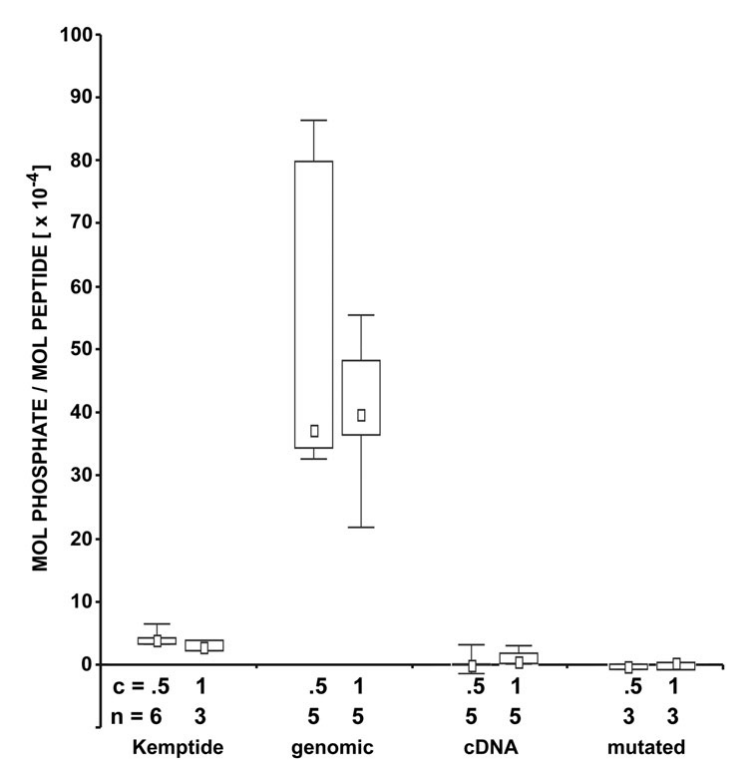
**In vitro phosphorylation of N-terminal undeca-peptides of *Drosophila *synapsin at two substrate concentrations**. The peptide encoded by the ''genomic'' sequence represents an excellent substrate for bovine PKA (compare with positive control ''Kemptide''). When a single amino acid of this peptide is modified by pre-mRNA editing (R4G) as encoded by the ''cDNA'', or when the two central serines are replaced by alanines (''mutated''), phosphorylation is abolished. Substrate concentrations (c) were 0.5 μg/μl and 1.0 μg/μl. Median, 25% – 75%, and Min-Max are shown. n indicates the number of independent measurements in each group. The difference in phosphate incorporation between genomic and cDNA peptides is significant at both substrate concentrations (p = 0.009).

In order to determine likely functional consequences of the editing described here, we performed in-vitro peptide phosphorylation experiments at two different substrate concentrations (Fig. 5). The N-terminal synapsin undeca-peptide containing the RRFS PKA recognition site encoded by genomic DNA is readily phosphorylated by bovine PKA, about 8 times faster than the positive control peptide (Kemptide), whereas no significant phosphorylation of the peptide containing the cDNA-encoded RGFS sequence is observed at the substrate concentrations used. Mutation to alanine of the two serines at position 6 and 7, which represent possible phosphorylation target amino acids of the genome-encoded N-terminal peptide, abolishes phosphorylation.

## Discussions and Conclusion

In this work we have investigated a discrepancy between the *Drosophila *synapsin cDNA sequence published earlier [26] and the BDGP sequence at the junction of exons 4 and 5 of the *synapsin *gene. In all wild-type lines examined we verified the genomic sequence of the genome project (AE003686) including the normal GT-AG splice consensus. Thus it seems unlikely that this region is polymorphic. The genomic sequence encodes the canonical PKA recognition motif RRxS in the A-domain of the *Drosophila *synapsin. This motif is also found in all other known synapsins. In vertebrates phosphorylation at this site apparently is involved in the redistribution of the protein during synaptic activity [25,13,14]. However, in *Drosophila *the genomic sequence reveals a single base pair difference to the published cDNA which encodes RGFS at the kinase recognition motif. Since the first canonical start codon (ATG) of the open reading frame of the *Drosophila synapsin *gene is located downstream of the conserved A domain and the kinase recognition site, we had earlier identified the amino acid sequence of the N-terminus of the 70 kDa synapsin isoform by Edman degradation of the immuno-affinity purified protein [27]. This independent data identified an unconventional leucine encoded by CTG as the first amino acid and clearly supported the cDNA encoded motif RGFS, strongly suggesting mRNA editing at this site.

To verify the cDNA sequence and to obtain a semi-quantitative measure of the efficiency of this editing we isolated mRNA of embryos/1^st ^instar larvae, 3^rd ^instar larvae, pupae, and heads and bodies of adults of different wild-type lines and the eye colour mutant *w*^*1118 *^which is frequently used to generate transgenic lines, and directly sequenced RT-PCR products in the region of interest. Surprisingly, in all samples except embryos/1^st ^instar larvae we found no trace of the genomic sequence, indicating that more than 90% of the pre-mRNA was edited, as estimated from the signal to noise ratio of the sequencing trace. (The presence of more than 10% unedited mRNA would have been detected as a double peak at one position, cf. asterisk in Fig. 4). Thus, the only major pool of primary transcripts that escapes editing is found very early in development. In two wild-type laboratory strains (WT Oregon-R and Canton-S) a certain fraction of the mRNA apparently was edited in addition at the first arginine codon of the kinase target motif. The resulting sequence GGFS presumably cannot be recognized by kinases.

**Figure 6:**
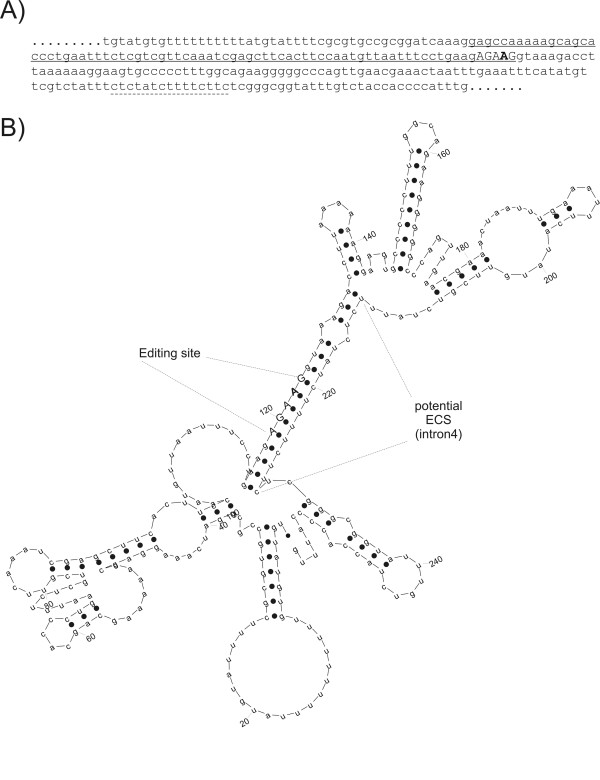
**Potential editing site complementary sequence (ECS) in intron 4 of the *synapsin *gene and predicted folding of the pre-mRNA**. (A) Genomic sequence of synapsin, exon 4 is underlined. Bases belonging to the codons for the two arginines in the protein kinase target motif are shown as capital letters, the edited adenosine is bold. The potential ECS is marked by the broken line in the sequence of intron 4. (B) Secondary structure of 250 bases of pre-mRNA of synapsin surrounding the editing site as predicted by the computer program MFOLD [35]. Capital and bold letters as in A.

All A to G discrepancies between genomic and cDNA in *Drosophila *investigated so far are due to the activity of the ADAR enzyme which catalyzes an adenosine-to-inosine conversion [8,9]. Expression of the *Drosophila *ADAR appears to be prominent in the nervous system. Interestingly, pre-mRNAs of several other proteins of the synaptic release machinery were also identified as A-to-I editing targets, such as synaptotagmin, dunc-13, stoned-B, complexin, and lap [8]. For hydrolytic deamination the enzyme needs a partial double-stranded RNA to form at the editing region. Normally this dsRNA is formed between the editing site and a complementary sequence in a neighbouring intron. Upon searching for a potential editing site complementary sequence (ECS) in the pre-mRNA of the *synapsin *gene of *Drosophila *we analysed 1 kb surrounding the kinase target site by a computer program which predicts secondary structures of an RNA molecule (MFOLD, [35]). In this analysis we detected a potential ECS region lying only 90 bp downstream of the edited arginine codon (Fig. 6). The ECS has a length of 15 bp similar to the size found e.g. in the ECS of the mammalian glutamate receptor GluR-B mRNA [3]. Thus the pre-mRNA of *Drosophila *synapsin can form a secondary structure containing a double helical stem that could make it a target for ADAR in the nervous system of *Drosophila*.

The new *Adar *mutant allele isolated here suffered a deletion of the entire first exon and 210 bp upstream sequences which presumably contain essential regulatory sequences, but the coding region remains intact. The fact that flies homozygous for this allele show a very similar phenotype to null mutants and are unable to edit the *synapsin *pre-mRNA suggests that this new allele is a severe hypomorph or a null allele.

With the methods used here we were unable to detect unedited mRNA in 3^rd ^instar larvae, pupae, and adults. Such high RNA editing efficiency in adult *Drosophila *has also been observed at four different editing sites of the L-type voltage gated calcium channel *Ca-alpha 1D *[37] and in substrates of the ADAR2 enzyme of mammals [38]. Possibly, unedited versions of these proteins are required earlier during development. This may also be true for *Drosophila *synapsin as we find unedited mRNA in embryos/first instar larvae. Like differential splicing, RNA editing is extensively used in *Drosophila *to generate protein diversity far beyond what is expected from the number of protein coding genes. Another evolutionary advantage of RNA editing may be the possibility to adjust the ratio of the abundance of two isoforms to any value between 0 and 1, rather than only to the 0, 1/2 or 1 possible by allelic encoding. So at first sight 100% editing would not seem to make much sense. However, if editing was reduced or absent in only a relatively small subset of neurons, we would not be able to detect this in our experiments. On the other hand, editing of the first arginine codon of the RRFS motif occurred only in two out of nine strains investigated and here only with about 50% efficiency. To show that the modification of the RNA sequence is restricted to the conserved N-terminal kinase target motif in the A-domain we also investigated the only other RRxS motif in *Drosophila *synapsin. Here no discrepancy between genomic DNA and cDNA sequences was observed. This result also represents an additional control against possible artefacts. Editing at the N-terminus was found in all laboratory strains and also in newly collected flies from different parts of central Germany. We conclude that the RNA editing described here is not an adaptation of inbred stocks to a laboratory environment that leads to degeneration of many adaptations that develop or are maintained under natural selection pressure.

We finally investigated likely functional consequences of the editing of synapsin described here. We measured the in vitro phosphorylation by bovine PKA of N-terminal undeca-peptides containing the edited, the unedited, and, as a negative control, a mutated amino acid sequence. These experiments clearly demonstrate that the peptide containing the genomic RRFS sequence represents an excellent substrate in this assay, while the edited version (RGFS) is not efficiently phosphorylated. Quantitative measurements of Michaelis-Menten constants should eventually be obtained for intact synapsin isoforms rather than on peptides, but such experiments are beyond the scope of this paper. However, the present in-vitro peptide phosphorylation data strongly suggest that editing also influences phosphorylation of synapsin by PKA in vivo. Since synapsin knock-out flies are impaired in learning and/or memory [27,28] we speculate that phosphorylation of *Drosophila *synapsin in the A domain is subject to cell-specific fine regulation by RNA editing. This now needs to be tested by appropriate phosphorylation assays using wild-type and transgenic flies with targeted mutations that prevent or simulate synapsin phosphorylation in conjunction with studies on the behavioural impairments of these flies as described for synapsin knock-out animals [27,28].

## Methods

### Flies

Flies were maintained in the laboratory at 25°C or 18°C under a 14/10 h light/dark cycle at 60–70% relative humidity. Wild-type strains Berlin, OregonR, CantonS, and Lindelbach, as well as the *white *stock *w*^*1118*^, were cultured under these conditions for more than 15 years. The wild-type lines Fulda, Westerwald, Schweinfurt and Bad Salzschlirf were collected in the fields. They were identified as species *Drosophila melanogaster *using the protocol provided by B. Shorrocks [39]. The P element insertion line w^1118 ^P{GT1}Adar^BG02235 ^that was used for a jump-out mutagenesis was generated by the Bellen lab and provided by the Bloomington *Drosophila *stock center at Indiana University.

### DNA preparation

50 flies were homogenized in 500 μl homogenization buffer [stock: 0.1 M NaCl; 0.1 M Tris HCl pH 8.0; 50 mM EDTA; 0.5% SDS; shortly before use 5.5 μl/ml RNase A (10 mg/ml) and 20 μl/ml Protease K (10 mg/ml) were added] and incubated for 30 minutes at 68°C. Next, 75 μl 8 M potassium acetate were added followed by standard phenol and EtOH extraction. Finally, the DNA pellet was resuspended in 50 μl TE buffer.

### RNA preparation

Total RNA was isolated by homogenizing 100 flies in 1 ml TRIzol (Life Technologies) followed by 5 minutes incubation at room temperature (RT). After adding 200 μl chloroform the samples were centrifuged (12,000 g) and the upper phase was selected for an isopropanol precipitation. The RNA was resuspended in 100 μl DEPC-water. The mRNA fraction was isolated using the Oligotex mRNA Mini Kit from QIAGEN.

### cDNA synthesis

cDNA was produced using oligo-dT-primers (MWG-Biotech AG) following the protocol of the "Omniscript" Reverse Transcriptase (QIAGEN). The samples were incubated for 1 hour at 37°C after which the enzyme was inactivated by incubation at 93°C for 5 minutes.

### Synapsin specific amplification

Genomic DNA or cDNA of the *synapsin *gene was amplified using different sets of primers (all from MWG Biotech AG):

DNA: forward-primer: TGT ATT TTC CGC TGC CGC; reverse-primer: TCG GCG CAC TGA CAC CAC.

cDNA: first PCR with forward-primer: GGG CAA ATA ACG AGG ACC, and reverse primer: TTG TCC TTG CTG AAT GCC; nested PCR with forward primer: CGG ATA GCC TGA GAT TCG, and reverse primer: GTC GGC TGA TCT TGG AT.

PCR was performed using standard protocols, followed by electrophoresis in 0.8–1.0% agarose gels. The PCR fragments were isolated from the gel using the QIAquick PCR purification kit from QIAGEN.

### Sequencing

DNA fragments were sequenced using the ABI PRISM™ *BigDye*™ Terminator Cycle Sequencing Ready Reaction Kit from ABI PRISM™ with the appropriate program in an Eppendorf gradient thermocycler. Sequence files were analysed using the program Chromas (Version 1.45).

### RNA structure

RNA secondary structure was analyzed using the MFOLD program [35,36].

### Mutagenesis of the Adar gene

In the line w^1118 ^P{GT1}Adar^BG02235 ^generated by the Bellen lab as part of the Berkeley *Drosophila *Genome Project, a modified P-element transposon is inserted in the first intron 9 bp downstream of the 5' exon-intron boundary, as was verified by PCR and sequencing. Females of this line were crossed to "jump-starter" males (Δ2–3^Ki, p^). 100 F1 males were crossed to *FM6a, w B *balancer females. The F2 generation was screened for females with white, kidney-shaped eyes (*Bar*). 300 lines were set up as balanced stocks from these individuals. Homozygous flies were subjected to PCR to characterize deficiencies produced by the P remobilization. The following primers were used:

*Adar *gene: Forward: AAG AGC AGC ACC GCA CC; reverse: ACC CCT TAT CCA CTA CCC

P{GT1}: Forward: CCG TTA CGC CAA CGA GG; reverse: GTC GGC AAA TAT CGC ATG C

The ends of the large deficiency in the *Adar *gene were identified by sequencing with the primers:

Forward: GGG GTA CAA TTT CCG CAA AG; reverse: GGT CGG GAC GGC AAG AT

### Peptide phosphorylation assay

The following N-terminal synapsin undeca-peptides were used as substrates:

genomic sequence: L-K-R-R-F-S-S-G-D-L-S

cDNA sequence: L-K-R-G-F-S-S-G-D-L-S

mutated (destroyed phosphorylation site): L-K-R-R-F-A-A-G-D-L-S

The peptides were synthesised by Biosynthan Gesellschaft für bioorganische Synthese mbH (Berlin; Germany). They were purified by HPLC (95% pure) and their identity was confirmed by MALDI-TOF mass spectroscopy. As positive control we used LRRASLG (Kemptide) (Bachem, Bubendorf, Switzerland). The substrates were resuspended in H_2_O at a final concentration of 20 μg/μl. The assay mixture for in vitro phosphorylation contained in a total volume of 50 μl the following (final concentrations): ATP 300 μM; 3 μCi γ^32^P ATP (10 μCi/μl; 3000 Ci/mmol) (GE Healthcare Life Sciences UK, LTD; Buckinghamshire; UK); 50 mM β-Glycerophosphate pH 7.3; 1.5 mM EGTA; 1 mM DTT; 10 mM MgCl_2_; 25 μg or 50 μg substrate and, to initiate the reaction, 100 ng bovine PKA (Biaffin GmbH & Co KG; Kassel; Germany). The mixture was incubated at 30°C for 4 minutes before the reaction was stopped by spotting the sample onto p81 phosphocellulose paper (diameter 2.5 cm; Whatman International LTD; Maidstone; UK). The papers were washed three times for 15 minutes in 175 mM H_3_PO_4 _to remove unbound ATP, before transferring them to scintillation vials containing 3 ml H_2_O. The amount of radioactive ^32^P incorporated into the substrate was determined in a scintillation counter (LKB wallac 1214 Rackbeta, Liquid Scintillation counter) by measuring the Tscherenkow radiation. Each of the four experimental conditions was tested five times, the controls between 3 and 6 times. Background levels, determined by omitting PKA from the mixture, were subtracted from the experimental values (leading in some cases to "negative" counts) and the resulting data were used to calculate phosphate incorporation stoichiometry for each peptide and concentration. As expected for low substrate concentrations, incorporation rates were not significantly different for 0.5 and 1.0 μg/μl substrate concentrations. Non-parametric Kruskal-Wallis test comparing the 8 groups reveals significant differences (p = 0.0001) and pair-wise comparisons between genomic and cDNA encoded peptides demonstrate significantly different phosphorylation rates at both substrate concentrations (p = 0.009, Mann-Whitney test, significance limit p < 0.025 due to Bonferroni's correction).

## Authors' contributions

SD conceived of the study, performed the majority of the experiments, and wrote a first draft of the manuscript, VN established and performed the peptide phosphorylation assay, UW carried out a large fraction of the molecular work, JH supervised the phosphorylation experiments, TZ supervised the mutagenesis experiment, EB detected the sequence discrepancy, supervised the work, participated in the design and coordination of the study, and wrote the final manuscript.
